# Overview of Research on Leishmaniasis in Africa: Current Status, Diagnosis, Therapeutics, and Recent Advances Using By-Products of the Sargassaceae Family

**DOI:** 10.3390/ph17040523

**Published:** 2024-04-18

**Authors:** Fatouma Mohamed Abdoul-Latif, Khadija Oumaskour, Nadira Abdallah, Ayoub Ainane, Ibrahim Houmed Aboubaker, Ali Merito, Houda Mohamed, Tarik Ainane

**Affiliations:** 1 Institut Supérieur des Sciences de la Santé (ISSS), Djibouti City P.O. Box 2530, Djibouti; 2 Medicinal Research Institute, Center for Studies and Research of Djibouti, IRM-CERD, Route de l’Aéroport, Haramous, Djibouti City P.O. Box 486, Djibouti; 3 Superior School of Technology of Khenifra, University of Sultan Moulay Slimane, P.O. Box 170, Khenifra 54000, Morocco; 4 Peltier Hospital of Djibouti, Djibouti City P.O. Box 2123, Djibouti

**Keywords:** Africa, *Sargassaceae*, diagnosis, leishmaniasis, natural product, treatment

## Abstract

Leishmaniasis in Africa, which has been designated as a priority neglected tropical disease by various global organizations, exerts its impact on millions of individuals, primarily concentrated within this particular region of the world. As a result of the progressively grave epidemiological data, numerous governmental sectors and civil organizations have concentrated their endeavors on this widespread outbreak with the objective of devising appropriate remedies. This comprehensive examination delves into multiple facets of this parasitic ailment, scrutinizing the associated perils, diagnostic intricacies, and deficiencies within the existing therapeutic protocols. Despite the established efficacy of current treatments, they are not immune to deleterious incidents, particularly concerning toxicity and the emergence of parasitic resistance, thus accentuating the necessity of exploring alternative avenues. Consequently, this research not only encompasses conventional therapeutic approaches, but also extends its scope to encompass complementary and alternative medicinal techniques, thereby striving to identify innovative solutions. A particularly auspicious dimension of this study lies in the exploration of natural substances and by-products derived from some brown algae of the *Sargassaceae* family. These resources possess the potential to assume a pivotal role in the management of leishmaniasis.

## 1. Introduction

Leishmaniasis, which has been categorized as a priority neglected tropical disease by the World Health Organization (WHO), presents a significant obstacle in the attainment of the Sustainable Development Goals (SDGs) by 2030 [1]. This particular parasitic infection, which exhibits a variety of clinical manifestations, is caused by different species of the *Leishmania* genus, resulting in complications that range from small skin ulcers to potentially fatal systemic damage to vital organs such as the bone marrow, liver, and spleen [2]. Leishmaniasis is prevalent in over 100 countries, affecting approximately 350 million individuals, with 12 million of them being infected, primarily in tropical and subtropical regions, particularly in Africa [3,4]. Thus, the impact of leishmaniasis on this continent is significant, often impacting vulnerable populations and being exacerbated by numerous risk factors, deficiencies in diagnostic approaches, and the absence of effective treatments [5,6,7].

The disease’s clinical manifestations take various forms, primarily visceral and cutaneous leishmaniasis, with less common variations influenced by genetic, geographical, environmental, and climatic factors [8,9,10]. The intricate process of host infection is influenced by a subtle interaction among the parasite species, its virulence factors, and the host’s immune response [11]. Consequently, the prevention of leishmaniasis predominantly centers on the control of vectors and their reservoirs, primarily sandflies, as well as the implementation of appropriate hygiene measures [12]. However, persistent challenges related to diagnosis and restricted access to healthcare in certain regions continue to impede optimal prevention [13]. Recently, initiatives have been devised to enhance the control and treatment of leishmaniasis, incorporating various innovations in traditional diagnostic techniques and the development of novel therapeutic approaches [14]. Current methodologies, which rely on parasitological, immunological, and molecular techniques, and incorporate the most up-to-date technological advancements, have significantly contributed to the enhancement of drug formulations, with a focus on targeted drug delivery and controlled release [15]. Current therapy for human leishmaniasis depends on a range of therapeutic drugs such as amphotericin B, antimonials, sitamaquine, pentamidine, paromomycin, and miltefosine. Nevertheless, these medications possess significant disadvantages, including toxicity, undesired side effects, the emergence of parasitic resistance, and often painful administration procedures [16,17,18].

In this particular context, our overview centers on the present condition of leishmaniasis in Africa, exploring risk factors, and recent and traditional advancements in diagnostic, preventive, and control strategies. We also investigate therapies, encompassing complementary and alternative medicinal approaches, dietary practices, and natural substances and by-products derived from brown algae of the *Sargassaceae* family, particularly the genera *Cystoseira* and *bifurcaria*. This research aims to offer a comprehensive overview of current diagnostic techniques and treatment choices, whilst delving into the potential of algae as therapeutic agents. It will provoke contemplation on innovative alternative approaches to combat Leishmania infection, envisioning a future where pioneering solutions will aid in mitigating this menace to global well-being.

## 2. Methods

The methodology adopted for this study focused on overview of leishmaniasis in Africa is based on a methodical and robust approach, encompassing the current status, therapeutic strategies, and promising initiatives. The major emphasis of this process lies in the innovation introduced by the processing of algae by-products of the genera Cystoseira and bifurcaria. This approach is broken down into three successive stages: collection of information, analysis, and use of specialized tools.

The first step, information gathering, involves a careful search for relevant data. This step requires the exploration of various sources and the methodical aggregation of the information obtained. The second phase, analysis, requires an in-depth evaluation of the collected data, highlighting the key specifications of the study implementation. The third stage is characterized by the judicious use of specialized tools, integrated strategically to refine the analysis and facilitate the understanding of the complexities inherent in this research.

**Figure 1:**
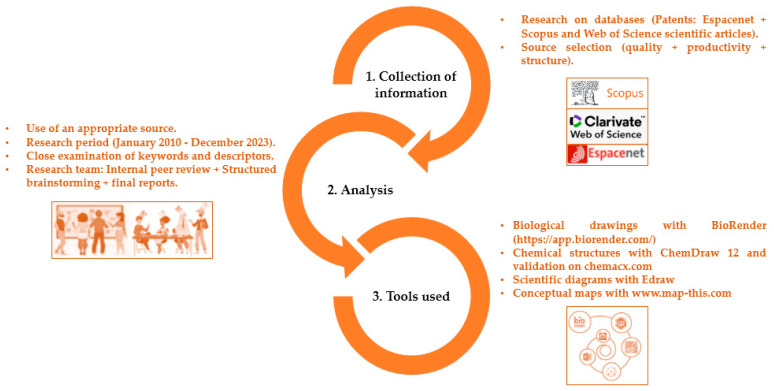
Work methodology.

The flowchart in Figure 1 provides a visual representation of the different steps of the methodological process, highlighting the logical sequence and interconnection of the components used in this study.

### 2.1. Collection of Information

One of the fundamental steps in composing an overview involves conducting targeted searches on pertinent databases that are associated with the process of gathering information. The process of data collection is inherently intertwined with the availability of specific information pertaining to the term “leishmaniasis”. Consequently, it is crucial to rely on dependable and appropriate sources of information to engage in comprehensive scientific exploration and construct effective arguments. As part of the patent search, the “Espacenet” database was utilized, which grants comprehensive access to critical information. Simultaneously, the Scopus and Web of Science databases were employed to search for scientific articles, offering comprehensive and reputable resources. These databases, which are presently accessible, enjoy widespread distribution within the scientific literature, thereby ensuring the pertinence and reliability of the extracted data. The selection of these databases was based on their reputable standing and ease of accessibility, thus contributing to the assurance of the good information gathered during all parts of this study.

### 2.2. Analysis

When conducting an assessment of scientific data obtained through a bibliographic search, it is imperative to take into account various indicators in order to ensure the quality and relevance of the sources. These indicators encompass the quality of impacts, productivity, and structural aspects. As previously stated, a suitable source was employed for the purpose of retrieving patents and scientific articles [19,20]. The time frame considered for this search extended from January 2010 to December 2023. Subsequently, a comprehensive examination of keywords and descriptors was undertaken, with a particular focus on highlighting the significance of treatments for leishmaniasis that utilize substances derived from brown algae belonging to the genera Cystoseira and bifurcaria. Following this procedural step, all bibliographic data were subjected to the scrutiny of peer review within the confines of our collaborative research team. This team, composed of team 1 from Djibouti, team 2 from Morocco, and team 3 (comprising two individuals from each country), convened to meticulously develop and discuss summary reports during structured brainstorming sessions.

### 2.3. Tools Used

Various tools, encompassing both software and online platforms, were utilized to optimize the process of scientific communication, enhance visual representation, and ensure the effective transmission of information by employing specific tools. For instance, BioRender (https://app.biorender.com/ accessed on 5 December 2023) was leveraged to facilitate the creation of biological drawings in an online format, thereby benefiting from the utilization of templates that exhibit superior quality. In order to establish chemical structures, the software ChemDraw 12 was employed, with the online interface of chemacx.com being utilized to verify the structural integrity of these chemical arrangements. Simultaneously, the versatile software Edraw 12 was deployed to proficiently generate scientific diagrams. Furthermore, the interactive online platform www.map-this.com accessed on 10 December 2023 was employed to produce concept maps, which are particularly conducive to scientific planning and learning.

## 3. Results and Discussion

### 3.1. Description of Leishmaniosis

Leishmaniasis is a disease transmitted by a vector, resulting from infection by a parasitic protozoan that is obligatory, belonging to the genus Leishmania within the family Trypanosomatidae [21]. The vector responsible for transmitting this disease is composed of female sandflies that are infected, specifically those of the Phlebotomus genus in the Old World and the Lutzomyia genus in the New World [22]. There are more than seventy different animal species, including humans, that can serve as natural reservoirs for the Leishmania parasites. Leishmaniasis is caused by protozoan parasites that belong to more than 50 distinct species of Leishmania, and the transmission of these parasites is facilitated by over 90 species of sandflies [23]. The disease presents itself in three primary forms. Visceral leishmaniasis (VL), also known as kala-azar, has a case fatality rate of over 95% if not treated. It is characterized by intermittent fever attacks, weight loss, hepatosplenomegaly (enlargement of the liver and spleen), and anemia [24]. Cutaneous leishmaniasis (CL) is the most prevalent form of the disease, causing changes in the skin, particularly ulcers, in exposed areas of the body. These ulcers can result in permanent scarring, which can lead to stigma and significant disabilities [25]. Mucocutaneous leishmaniasis leads to partial or complete destruction of the mucous membranes in the nose, mouth, and throat [26].

**Figure 2:**
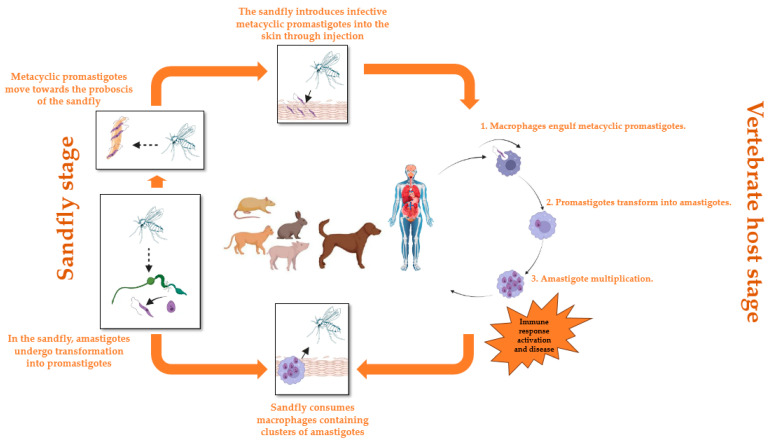
Life cycle of Leishmania parasites.

The life cycle of the Leishmania parasite commences when the female sandfly partakes in a blood meal from an infected vertebrate host, thereby ingesting macrophages or other host cells containing amastigotes, which are the intracellular form of the parasite. Once inside the sandfly, the amastigotes undergo a transformation into promastigotes within the insect’s midgut, where they undergo multiplication and migration towards the anterior region of the intestine, attaching themselves to the intestinal wall. After a few days, the promastigotes differentiate into infectious and highly mobile metacyclic promastigotes. These metacyclic promastigotes proceed towards the proboscis of the sandfly, poised to be transmitted to a new vertebrate host. When an infected sandfly feeds on the blood of a vertebrate host, it introduces the infectious metacyclic promastigotes into the skin. Inside the host, the metacyclic promastigotes are engulfed by phagocytes, such as macrophages, and undergo development into amastigotes, which represents the intracellular stage of the parasite. The amastigotes experience multiplication within host cells, predominantly within macrophages, resulting in the formation of clusters referred to as parasitophorous vacuoles that are loaded with amastigotes. The presence of Leishmania parasites stimulates an immune response, ultimately leading to the clinical manifestations of leishmaniasis. If an infected sandfly feeds on the blood of an already infected vertebrate host, the cycle is initiated anew as it ingests the amastigote-laden macrophages (Figure 2) [27,28,29].

### 3.2. Overview of Leishmaniasis in Africa

Leishmaniasis, a parasitic disease caused by the Leishmania parasites, is highly prevalent in various regions across the globe, spanning Africa, Asia, Europe, and the Americas. The incidence of this pathology is influenced by a multitude of complex and interconnected parameters, including climate, environment, and socio-economic conditions. These factors interact with each other and contribute to the spread and persistence of the disease. According to the data obtained from the World Health Organization (WHO) in 2022, the number of reported new cases of leishmaniasis exceeds 500,000 annually, leading to a staggering number of deaths, almost reaching 10% [30,31,32].

In the African continent, the frequency of leishmaniasis varies significantly, ranging from 14% to 50% in different regions. This wide range of prevalence categorizes leishmaniasis as endemic in many countries across the continent, particularly in East Africa, North Africa, and certain areas of South Africa. The distribution of the disease within Africa is not uniform, and the frequency of different species of Leishmania can exhibit variations depending on the specific region. Among the identified species, *Leishmania donovani* and *Leishmania infantum* are the most frequently responsible for causing leishmaniasis in Africa. Countries such as Ethiopia, Somalia, Sudan, and South Sudan report alarmingly high rates of visceral leishmaniasis, a severe form of the disease that affects multiple organs, while Algeria experiences a notable percentage of cutaneous leishmaniasis cases, which primarily affects the skin [33,34,35].

Despite the availability of data on leishmaniasis in Africa, it is important to acknowledge that more than half of the African nations lack accurate and comprehensive information regarding the extent and impact of the pandemic. Insufficient resources and limited access to healthcare facilities contribute to the challenges faced in accurately assessing the prevalence of leishmaniasis in Africa. Consequently, there is a strong suspicion that the actual burden of the disease is significantly higher than what is currently reported, suggesting a widespread underdiagnosis and underreporting of leishmaniasis in several areas [36,37].

### 3.3. Risk Factors

**Figure 3:**
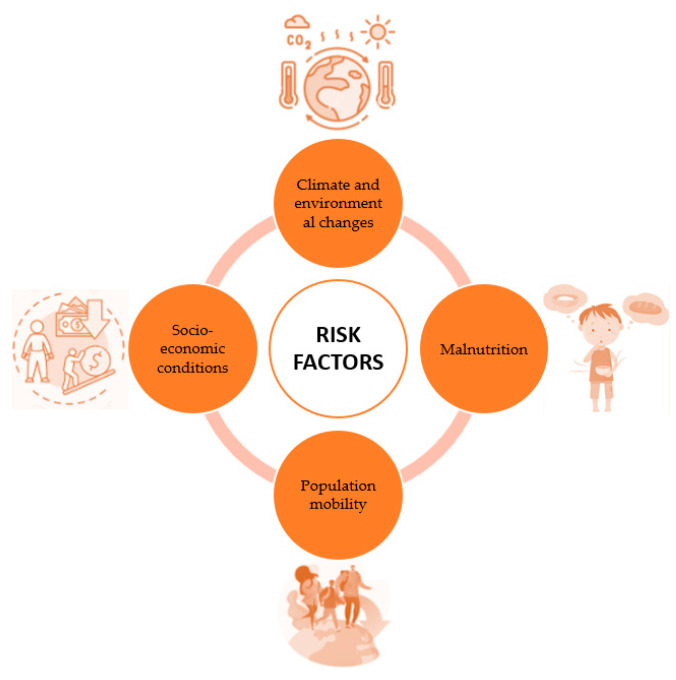
Main risk factors.

The risk factors associated with leishmaniasis encompass a complex array of dynamics (Figure 3), wherein climate and environmental changes emerge as the primary determinants. The expansive urbanization and deforestation, along with the increasing human and animal activities in forested areas, constitute the backdrop against which these risks materialize. The significant alterations in the environment directly impact the incidence of leishmaniasis. The vagaries of the climate, characterized by fluctuations in temperature and precipitation, influence the size and geographical distribution of sandfly reservoirs, which serve as vectors for transmitting the Leishmania parasite. Climate change also exerts adverse effects beyond mere weather variations, impacting crucial aspects such as population mobility [38]. Droughts, famines, and floods induce massive migrations of both humans and animals to areas where the transmission of the parasite becomes alarmingly prevalent. These movements further exacerbate the spread of the disease and contribute to the formation of epidemic outbreaks. Another key determinant of leishmaniasis risks lies within the spectrum of malnutrition. Deficiencies in dietary protein, iron, vitamin A, and zinc serve as predisposing factors that increase the likelihood of the infection progressing into severe forms of the disease. Malnutrition, by compromising the immune system, creates a fertile ground for the development and progression of the infection [39]. Population and animal mobility emerge as critical factors in the leishmaniasis scenario. Epidemics of the disease frequently arise as a result of massive movements of people and animals to areas where the transmission of the parasite is particularly active [40]. Furthermore, socio-economic conditions also trigger dynamics that facilitate the spread of leishmaniasis. Poverty, as a risk factor, manifests through unsanitary housing and inadequate sanitation infrastructure, creating favorable conditions for the reproduction of sandflies. These vectors, attracted to crowded environments, increase the likelihood of contact with human populations, thereby contributing to the dissemination of the disease. Certain human behaviors, such as sleeping in the open air or on the ground, also serve as variables that can heighten the risk of exposure to leishmaniasis [41].

### 3.4. Diagnosis of Leishmaniasis

**Figure 4:**
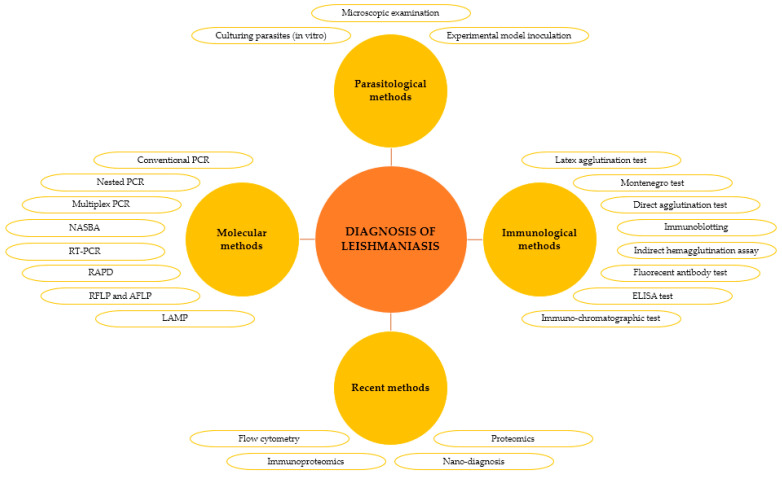
Conventional and recent diagnostic methods of leishmaniasis.

The initial diagnosis, imperative for the adequate treatment, cure, and eradication of diseases, relies on various methodologies for leishmaniasis, ranging from parasitological techniques to immunological and molecular approaches, integrating the latest technological advances. Figure 4 illustrates methods for diagnosing leishmaniasis.

#### 3.4.1. Parasitological Diagnosis

The parasitological diagnosis of leishmaniasis is mainly based on three methods: microscopic analysis, in vitro culture, and inoculation in an experimental model. Microscopic analysis allows the detection of the amastigote form of the parasite in tissues, using dyes such as Giemsa, Panotic, and Wright. In vitro culture isolates the promastigote form of the parasite by culturing the parasites, while inoculation in an experimental model involves introducing the parasite into an animal model, usually mice, to observe the development of the disease. Although these methods are widely used due to their simplicity and moderate cost, they only allow the detection of parasites at the genus level, without distinction at the species level [42].

#### 3.4.2. Immunological Diagnosis

Immunological techniques offer a rapid, non-invasive approach to detect Leishmania antigens or antibodies in human patients. They include the latex agglutination test, Montenegro test, direct agglutination test, indirect immunoblotting, hemagglutination test, fluorescent antibody test, ELISA test, and immunochromatographic test. The Montenegro test, based on the type IV hypersensitivity reaction, offers an economical and sensitive approach, despite sometimes negative results at the start of visceral infection. The latex agglutination test relies on the agglutination of latex particles sensitized with leishmanial antigens, with high sensitivity and potential variability in specificity. The economical and semi-quantitative direct agglutination test (DAT) uses trypsin-treated promastigotes as antigens, demonstrating high sensitivity and specificity, although positivity persists after clinical recovery. Indirect immunoblotting offers high sensitivity and specificity, based on the transfer of Leishmania antigens to a membrane and their reaction with patient serum. The fluorescent antibody test (IFA) is rapid and accurate, distinguishing present infection from clinical cure, but requiring specific facilities. The indirect hemagglutination assay uses human erythrocytes sensitized to Leishmania antigens, with high values of sensitivity and specificity varying depending on host immune responses. ELISA, because of its high sensitivity and specificity, remains a major method, although the choice of antigen can influence sensitivity, requiring well-equipped facilities. The immunochromatographic test (ICT) using the rK39 antigen constitutes a rapid, economical, and non-invasive screening test for visceral leishmaniasis, remaining effective for large-scale screening despite variations in performance between epidemiological populations [43].

#### 3.4.3. Molecular Diagnosis

With the advent of molecular biology techniques, the diagnosis of leishmaniasis has undergone a significant transformation. These molecular methods offer an effective alternative to conventional parasite detection, with increased sensitivity and specificity, as well as the ability to analyze large numbers of samples simultaneously. Various molecular techniques have been developed for the identification, detection, quantification, and ethnological analysis of Leishmania. Among these, PCR-based tests have become the standard in molecular diagnostics for researchers and healthcare professionals. Conventional PCR, a rapid and versatile in vitro approach, selectively amplifies the target DNA or RNA sequence, although it has disadvantages such as the need for trained expertise, the risk of contamination, and the inability to differentiate current infection from relapse after treatment. Nested and semi-nested variants of PCR have significantly improved sensitivity and specificity by reducing nonspecific DNA amplification, but they require precautions against contamination. Real-time PCR (RT-PCR), a modified method of PCR amplification, allows the simultaneous amplification and quantification of the target DNA molecule, although it is not species-specific. Multiplex PCR, amplifying multiple DNA targets simultaneously, improves sensitivity but is not recommended for routine use due to high costs. Nucleic acid sequence-based amplification (NASBA), using RNA polymerase to produce RNA and reverse transcriptase, provides superior sensitivity but does not allow the differentiation of Leishmania species. The methods of PCR-RFLP (restriction fragment length polymorphism) and AFLP (amplified fragment length polymorphism) are distinguished by the digestion of amplified DNA with restriction endonucleases and the analysis of the generated fragments, providing increased sensitivity, in particular for samples with low parasite load, and allowing the precise genomic analysis of different *Leishmania* spp., including the distinction between closely related strains [44].

### 3.5. Recent Diagnosis

Recent advances in the diagnosis of leishmaniasis have led to the emergence of sophisticated techniques. Notably, innovative approaches such as flow cytometry, proteomics, immunoproteomics, and nano-diagnostics have attracted attention. Among these techniques, flow cytometry has established itself as a practical and precise method for the diagnosis of visceral leishmaniasis, offering rapid and reproducible clinical applicability. Proteomics, focused on the analysis of protein complexes, uses mass spectrometry to assess protein abundance, post-translational modifications, protein interactions, and the structure and distribution of proteins within cells. In contrast, immunoproteomics aims to identify proteins linked to infection and stimulating immune responses. It uses techniques such as two-dimensional electrophoresis, Western blotting, and mass spectrometry. These methodologies have facilitated the discovery of specific biomarkers for leishmaniasis. Advanced molecular techniques, such as loop isothermal amplification (LAMP) and multilocus sequential typing (MLST), have brought significant improvements in the field of diagnosis. Finally, nano-diagnostics, merging nanoscience and nanotechnology, uses nanoparticles to detect Leishmania promastigotes. This approach offers a quick and economical method. These groundbreaking diagnostic innovations demonstrate increased sensitivity and specificity compared to traditional methods. They represent a crucial step forward in the fight against leishmaniasis [45,46,47].

### 3.6. Prevention and Control

**Figure 5:**
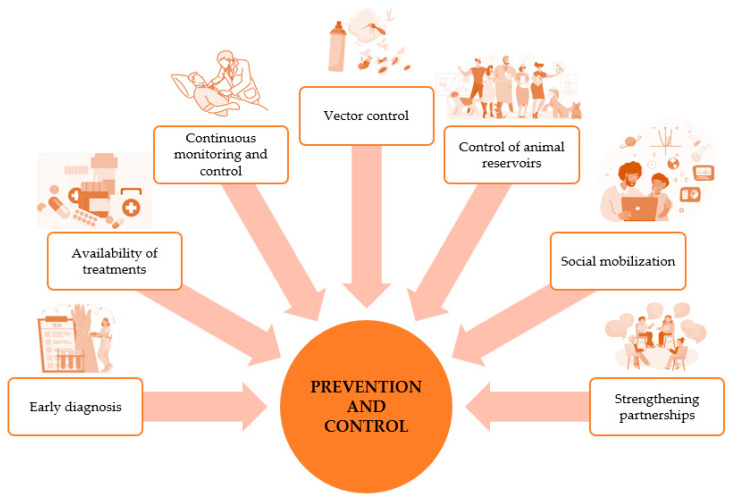
Main multidimensional approaches for the prevention and control of leishmaniasis.

The complex management of leishmaniasis and the need to prevent its spread requires a variety of tools and integrated strategies (Figure 5) [48]. A multidimensional approach is essential for effective prevention and control. The early detection of leishmaniasis and the rapid and effective implementation of treatment are fundamental pillars aimed at reducing the prevalence of the disease, preventing disability and death, and stemming transmission. At the same time, the continuous monitoring of disease burden and disease spread is crucial. Highly effective and safe drugs, particularly against various forms of leishmaniasis, are available, although their administration can present challenges [49]. Vector control, as a key component, aspires to mitigate or even interrupt disease transmission by reducing the sandfly population. Methods include insecticide spraying, the use of insecticide-treated bed nets, environmental management, and the promotion of personal protection. Active disease surveillance is imperative to enable rapid monitoring and appropriate interventions in the event of outbreaks or high case fatality rates under treatment [50]. The complexity of the fight against animal reservoirs requires an approach adapted to the local situation. Social mobilization and strengthening collaborations between governmental and non-governmental sectors are essential elements in the fight against leishmaniasis. Awareness and education activities within communities, aimed at changing behavior through effective interventions, must constantly adjust to the local situation. Building partnerships and collaboration with various stakeholders, as well as other vector-borne disease control programs, remains critically important for a comprehensive and integrated approach [50,51,52].

### 3.7. Treatment of Leishmaniasis

#### 3.7.1. Conventional Antileishmanial Therapy

**Figure 6:**
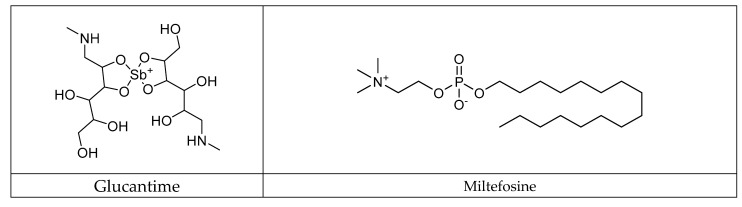

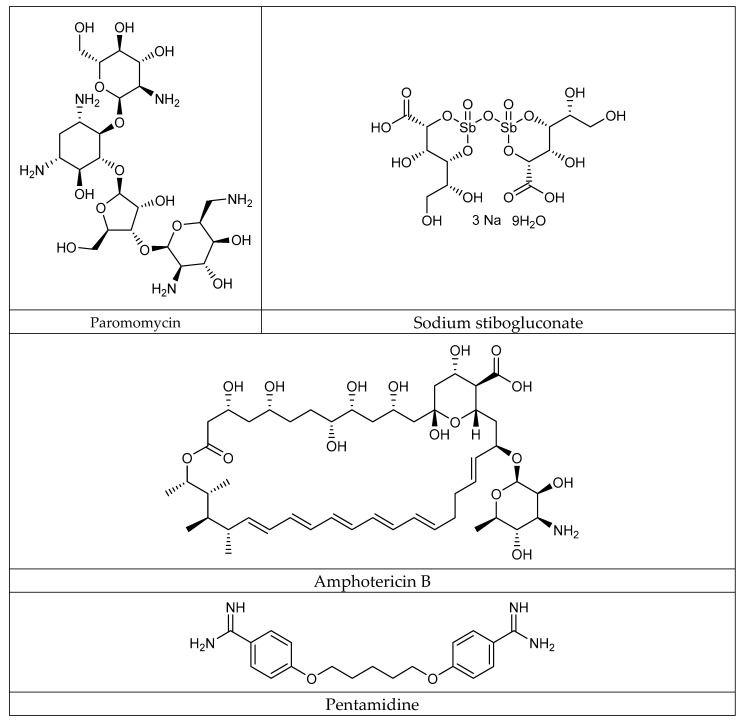

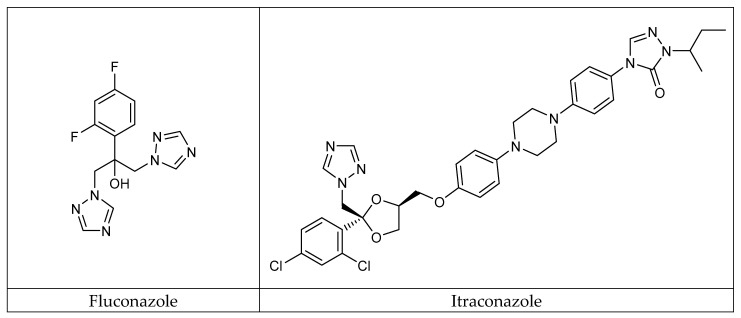
Main drug treatments for leishmaniasis.

The conventional treatment of leishmaniasis relies on the administration of varied therapeutic agents, each characterized by distinct mechanisms of action and specific considerations. Pentavalent antimoniate, used on the front line since 1945 for the clinical cure of the main forms of leishmaniasis, is available in two predominant formulations: meglumine antimoniate (Glucantime; Sanofi-Aventis^®^, Paris, France) and sodium stibogluconate (Pentostam. GlaxoSmithKline^®^, Minato, Tokyo) [53]. Identified as inactive in its pentavalent form, it requires reduction to its trivalent form to express its biological effectiveness. Potential mechanisms include reduction by thiols or thiol-dependent reductase, leading to the inhibition of the parasite virulence factor trypanothionin. It can also disrupt energy metabolic pathways, induce oxidative stress, cause DNA fragmentation, and lead to cellular apoptosis. Amphotericin B, an antifungal antibiotic, is positioned as a significant alternative, particularly in cases of resistance to antimoniate [54]. Its mechanism involves binding to the ergosterol of the fungal membrane, altering membrane fluidity and inducing cell death. However, amphotericin B deoxycholate is associated with major side effects such as nephrotoxicity, frequently requiring prolonged hospitalization. Liposomal formulations have been developed to mitigate toxicity while improving pharmacokinetics. Miltefosine, an oral medication, stands out as the only oral treatment for leishmaniasis [55]. Its mode of action involves the modification of cell membrane composition, damage to mitochondria, and the induction of programmed cell death. Although effective, its use is limited due to increasing resistance, toxic effects, and teratogenicity, restricting its administration in pregnant women. Paromomycin, an antibiotic from the aminoglycoside class, acts by inhibiting protein synthesis and disrupting the cell membrane. Approved for the treatment of visceral leishmaniasis in India, it nevertheless presents side effects such as pain at the site of infection, and liver, kidney, and ear toxicity. These diversified therapeutic approaches reflect the complexity of leishmaniasis management, highlighting the need for varied strategies to combat this disease. Furthermore, other antileishmanial therapies, such as pentamidine, have been studied for their action against the parasite [56]. Since its first synthesis in the 1940s, pentamidine has been explored due to cases of antimoniate resistance in the Indian subcontinent. However, its use has been abandoned due to toxicity, including pancreatitis leading to diabetes mellitus, hypoglycemia, hypotension, cardiac alterations, and hyperkalemia. On the other hand, azole antifungal agents such as fluconazole [57] and itraconazole [58] have been evaluated as therapeutic strategies, mainly for cutaneous forms. In the case of fluconazole administered orally for cutaneous forms, cure rates approach 60%, with a significant increase in effectiveness when the concentration of the drug is doubled. However, this treatment is associated with side effects such as hepatotoxicity and cardiotoxicity (Figure 6).

#### 3.7.2. Recent Advances in the Treatment of Leishmaniasis

Recent advancements in the treatment of leishmaniasis have garnered a growing interest in innovative therapeutic methods. Among these, combination therapy, drug repurposing, and nanotechnology are emerging as promising strategies aimed at optimizing the efficacy and customization of the treatment for this parasitic disease.

- Synergetic polytherapy

The observation of increasing drug resistance and relapse rates in conventional treatments for leishmaniasis has prompted the exploration of alternative solutions. Combination therapy, which has demonstrated its effectiveness in treating tuberculosis, malaria, and HIV, is emerging as a promising approach for leishmaniasis. The primary objective is to combine multiple drugs in order to reduce treatment duration, relapse rates, dosage, and fatal side effects, while also addressing drug resistance concerns [59]. Recent studies have evaluated the effectiveness of combining drugs such as itraconazole (an antifungal), ezetimibe (a lipid-lowering agent), and miltefosine [60,61]. These combinations have shown additive effects, significantly reducing the parasite load in the liver and spleen. Additionally, combination approaches involving the use of tamoxifen with meglumine antimoniate and liposomal Amp B with tretazicar have demonstrated promising results [62,63]. Clinical trials have assessed the combined use of AmBisome and miltefosine, revealing encouraging outcomes in immunocompromised patients co-infected with HIV and visceral leishmaniasis [64,65].

- Medication reassignment

Drug repurposing, also known as drug repositioning, is an innovative approach to identify new applications for clinically approved drugs that were originally developed for other conditions. This strategy offers significant advantages in terms of cost and development time reduction, as well as minimizing side effects and relapses. In the context of leishmaniasis, several drugs have been successfully repurposed. Antifungals such as Amphotericin B, miltefosine, and azoles (fluconazole, itraconazole, and posaconazole) have demonstrated promising efficacy [66]. Furthermore, antidepressants like sertraline have been evaluated and shown significant leishmanicidal action with a multi-target mechanism of action [67]. In silico studies have identified several drugs, including rifabutin, lamivudine, and metformin, as having the potential to eradicate the Leishmania parasite [68,69,70].

- Nanotechnology

Nanotechnology is emerging as a promising avenue in the treatment of leishmaniasis. By utilizing nanomaterials such as liposomes, metal nanoparticles, and polymeric derivatives, nanotechnology offers a platform for targeted drug delivery, enhanced drug solubility, and reduced side effects [71]. Nanoparticles are particularly well suited for targeting macrophages, which are key cells in leishmaniasis. Liposomes and polymeric nanoparticles can transport drugs directly to infected cells, thereby improving therapeutic efficacy [72]. Studies have confirmed that the utilization of nanoparticles for drug delivery induces leishmanicidal mechanisms, such as the increased generation of free radicals, the disruption of the mitochondrial membrane, and the suppression of the enzyme trypanothionine reductase [73].

#### 3.7.3. Drug Discovery Process

Despite the availability of some drugs suitable for leishmaniasis, the diversity of species, particularly in Africa with its specific climatic characteristics, constitutes a major challenge. The search for new drugs is imperative. The complex and expensive drug discovery process has generated strategies to optimize time and resources. These challenges underline the crucial importance of innovation to improve therapeutic effectiveness and fight against the variety of leishmaniases.

Key strategies in the drug discovery process:

- Fragment-based drug discovery

This approach uses specially designed molecules with automated techniques such as high-throughput crystallography to identify and optimize small molecules binding to target proteins. Techniques such as hydrogen/deuterium exchange coupled with mass spectrometry (HDX-MS) and fragment libraries play a crucial role in this strategy [74].

- Direct screening of the target

Based on the repurposing or modification of existing molecules, this process uses gene family platforms, compound libraries, computational models, and cellular and biochemical assays to evaluate the repurposing of existing molecules as a treatment for a different disease [75].

- Phenotypic drug discovery

Exploring biological systems or physiologically relevant cellular pathways, this approach uses advanced methods such as high-content imaging, advanced computing, advanced cellular assays, stem cells, SCORE, in vivo imaging, and zebrafish models. In the context of kinetoplastids, phenotypic methods adapt to the complex life cycles of parasites, requiring specific approaches for different hosts and life stages. Notably, for trypanosomatids, the discovery process generally targets the infectious form in the mammalian host, while for *Leishmania* sp., it focuses on the intracellular amastigote. This choice of life stage significantly impacts the results of the drug discovery process due to the adaptability of parasites to various environmental conditions [76].

### 3.8. Complementary and Alternative Medicine, and Dietary Patterns in Prevention and Supportive Leishmaniosis Care

CAM approaches have garnered considerable interest in African nations due to the socio-economic vulnerabilities that characterize these regions. In light of these realities, individuals afflicted with leishmaniasis are turning to alternative therapies, exploring various modalities such as aromatherapy, homeopathy, acupuncture, and herbal medicine [77]. The medicinal plants’ antileishmanial properties offer a promising avenue for managing the cutaneous symptoms associated with leishmaniasis, with decades of research highlighting the potential of specimens like *Artemisia annua*, *Plumbago scandens*, *Physalis angulata*, *Phyllanthus amarus*, *Piper aduncum*, *Peschiera (Tabernaemontana) australis*, and *Kalanchoe pinnata* [78].

Preliminary studies also suggest that acupuncture may enhance the immune system in infected patients. Nonetheless, this research remains limited, necessitating thorough evaluation to ascertain the efficacy and safety of these approaches, thus solidifying the scientific foundation for their dependability.

Conversely, the importance of tailored diets proves indispensable in leishmaniasis prevention and patient support. Investigations have underscored nutritional deficiencies among affected individuals, which directly impact the immune response. Diets rich in zinc, vitamin C, and protein are identified as significant contributors to strengthening the immune system, thereby enhancing the body’s ability to combat infection [79,80]. Implementing well-balanced diets also yields substantial benefits in mitigating the side effects of medical treatments. A comprehensive analysis of dietary patterns in the context of leishmaniasis prevention highlights the nutritional challenges faced by developing countries in Africa. Deficiencies in nutritional intake, particularly micronutrients and macronutrients, underscore the barriers these communities encounter [81]. Serum levels confirm the decline of the essential element zinc in leishmaniasis patients, emphasizing the need to pay specific attention to zinc inhibitors in their diet, including phytates [82]. This targeted focus on nutritional aspects, as indicated by dietary models, could play a pivotal role in the formulation of more effective prevention strategies.

### 3.9. Natural Products for Leishmaniosis Treatment

The utilization of natural substances in the therapy of leishmaniasis presents a significant potential, capitalizing on all residual biomasses, encompassing unrefined extracts, segments derived from diverse botanical and zoological components, as well as secondary metabolites. Throughout historical times, unrefined extracts from diverse botanical components, such as leaves, roots, seeds, fruits, and stems, have been acknowledged as remedies [83]. Asparagus gracilis, Stellaria media, Sida cordata, and Jurinea dolomiaea are examples of plants that have undergone rigorous evaluations regarding their efficacy against Leishmania tropica [84,85,86]. The segments derived from these plants have demonstrated noteworthy outcomes, displaying remarkable antileishmanial activities. Analogous investigations have been focused on 16 Brazilian medicinal plants, where the hexane segments of Dipteryx alata, the ethanolic extracts of Hymenaea stignocarpa, and the chloroform and ethanolic segments of Jacaranda cuspidifolia were identified as the most potent against *L. amazonensis* [87,88]. Additionally, the essential oils of Physalis angulata and Tetradenia riparia have exhibited promising outcomes in antileishmanial trials, thereby illustrating the therapeutic potential of these natural substances in leishmaniasis treatment [89,90].

Furthermore, alkaloid compounds are emerging as promising candidates for leishmaniasis treatment due to their frequently alkaline nature, resulting from specific amino acids, thereby generating a multitude of diverse structures. Alkaloids such as solamargine and solasonine, derived from Solanum lycocarpum, have shown remarkable activity both in vitro and in vivo against *L. mexicana*, surpassing the IC50 values of meglumine antimoniate [91]. Additionally, these compounds have regulated the immunochemical pathways of host cells, thereby enhancing the elimination of parasites. The efficacy of combination therapy has been conclusively established, particularly with the combinations of piperine and capsaicin against *L. infantum* [92]. Moreover, the utilization of nanoformulations, such as HDGG-AmB-Pip Nanoparticles, has improved the effectiveness of amphotericin B, thereby demonstrating promising outcomes both in vitro and in vivo [93]. Other alkaloids, including berberine, have also demonstrated leishmanicidal activity by inducing an imbalance in redox and impacting the mitochondria of parasites. Innovative formulations, such as CS-PEO-Berberine nanofibers, have exhibited promising results, thus suggesting topical alternatives for the treatment of cutaneous leishmaniasis [94].

On the other hand, current research is investigating the therapeutic effects of metabolites derived from the shikimate pathway, including flavonoids, lignans, neolignans, coumarins, quinones, and caffeic acid from vascular plants, against leishmaniasis. Flavonoids, resulting from the combination of the shikimate and acetate pathways, display a wide range of structures. These naturally occurring compounds have demonstrated specific antileishmanial activities against certain strains, particularly those affecting trypanosomes. For example, quercetin and apigenin, both flavonols extracted from Kalanchoe pinnata, illustrate the potential effectiveness of flavonoids against leishmaniasis by exhibiting significant activity against promastigotes and intracellular amastigotes of *Leishmania amazonensis* [95,96]. Lignans and neolignans, found in approximately 60 plant families, are phenylpropanoid compounds with properties that make them suitable for drug development. Research has shown the effectiveness of specific lignans, like dehydrodieuginol, against *L. amazonensis* promastigotes. Furthermore, coumarins, derived from hydroxyl groups, possess diverse structures that enable a range of biological activities, including anti-Leishmania effects [97]. Extracts from Calophyllum brasiliense have demonstrated good activity against promastigotes and intracellular amastigotes of *L. amazonensis* [98]. Caffeic acid, representing hydroxycinnamic acids, holds promise for structural modification to optimize the biological properties of its analogues. The radicals displayed inhibitory properties against Leishmania [99]. Moreover, considerable interest has been shown towards quinones, which are classified as benzoquinones, anthraquinones, and naphthoquinones, as antileishmanial agents. Benzoquinones, such as ubiquinone, participate in respiratory chain reactions [100]. Anthraquinones, dyes, and naturally occurring naphthoquinone exert biological effects on various pathogens, including *Leishmania* spp. [101].

Compounds derived from the mevalonate pathway have emerged as effective agents in the treatment of leishmaniasis, with a particular focus on terpenoids. Terpenoids, such as artemisinin obtained from *Artemisia annua*, have demonstrated activity against different species of Leishmania, showing notable effectiveness against *Leishmania major* [102]. Additionally, artemisinin has been proven effective against *L. donovani*, specifically targeting intracellular amastigotes [103]. Likewise, the compound (-)-α-bisabolol has exhibited significant activity against intracellular amastigotes of *L. infantum* and *L. donovani* [104]. The triterpene acids oleanolic and ursolic have shown efficacy against *L. amazonensis*, inducing programmed cell death and reducing the size of skin lesions in animal models [105]. Furthermore, innovative strategies, such as the use of nanocarriers, have been employed to enhance the bioavailability of ursolic acid, yielding promising results in vitro and in vivo. In the exploration of marine sources, terpenoid compounds from the sponge *Dendrilla membranosa* and the octocoral coral *Plumarella delicatissima* have also demonstrated promising leishmanicidal activity [106,107].

### 3.10. Use of Cystoseira and Bifurcaria Algae By-Products in Leishmaniosis Treatments (In Vitro)

Sargassaceae, the dominant family among the Fucales, recently emerged from the merger of the ancient Sargassaceae and Cystoseiraceae, representing a family polyphyly among brown algae [108]. This family, characterized by a diversity of genera, notably Cystoseira and Bifurcaria, has been the focus of intensive research since 1973. These studies have led to the discovery of a multitude of new bioactive compounds. For example, Valls and Piovetti synthesized 134 new diterpenoids isolated from ancient Cystoseiraceae up to January 1995 [109], while de Sousa et al. (2017) [110] as well as Gouveira et al. (2013) [111] compiled secondary metabolites from various Cystoseira species from 1995 to 2016. In parallel, Muñoz et al. (2013) [112] examined linear diterpenes from *Bifurcaria bifurcata*, shedding light on their biosynthetic pathways, biological activities, chemotaxonomy, and ecology.

**Figure 7:**
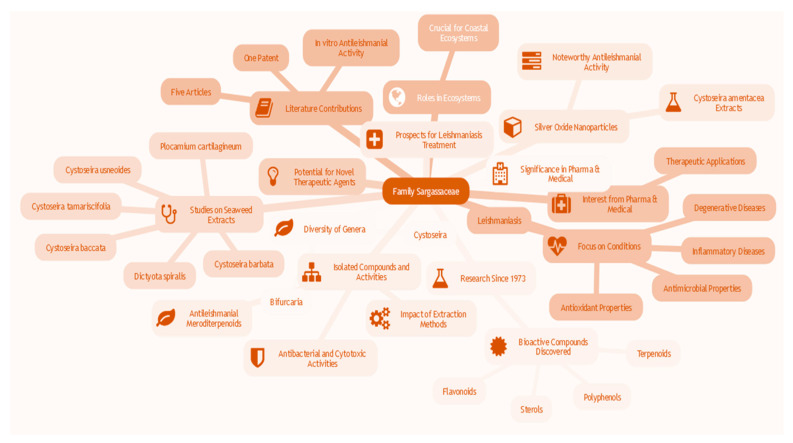
Mind map of the use of Cystoseira and bifurcaria algae by-products in leishmaniasis treatments (created with www.map-this.com accessed on 10 December 2023).

The genera Cystoseira and Bifurcaria are a classification of brown algae that belongs to the family *Sargassaceae* and is widely found in temperate marine African seas. These macroalgae form intricate colonies and play a crucial role in coastal ecosystems, contributing to biodiversity and ecological equilibrium [113]. The pharmaceutical and medical fields have become increasingly interested in both genera due to their abundant chemical composition, which includes various secondary metabolites such as terpenoids, flavonoids, sterols, and polyphenols [114]. These bioactive compounds have exhibited promising biological properties, including anti-inflammatory, antioxidant, antimicrobial, and antiviral activities [115]. The primary goals of pharmaceutical and medical exploitation of the genera Cystoseira and Bifurcaria lie in the investigation of their active compounds as potential sources of novel therapeutic agents. Research focuses on the discovery of molecules that can effectively treat a range of conditions, from parasitic infections like leishmaniasis to inflammatory and degenerative diseases. Recent studies have highlighted the antileishmanial properties of these algae, emphasizing their specific potential for the development of drugs against parasitic diseases [116]. Moreover, the medical exploitation of the genera Cystoseira and Bifurcaria also encompasses research aimed at harnessing their antimicrobial properties in the battle against infections, as well as their antioxidant potential for the prevention of diseases associated with oxidative stress. Ongoing studies are currently exploring the utilization of Cystoseira and Bifurcaria algae by-products in the treatment of leishmaniasis. This report critically examines a patent and five articles that elucidate the antileishmanial properties of products derived from various species of algae belonging to the Sargassaceae family. In terms of innovation, only one patent has been granted for the advancement of algae from the genera cystoseira and Bifurcaria (Figure 7).

The patent by Ainane et al. (2012) [117] delineated the utilization of substances derived from the brown alga *Bifurcaria bifurcata* in the management of leishmaniasis. The substances attained through Soxhlet extraction utilizing solvents of escalating polarity, such as hexanic, ethereal, and chloroform, exhibited noteworthy in vitro antileishmanial activity. The inhibition concentrations (IC50) of the hexanic, ethereal, and chloroform extracts were 46.83 μg/mL, 51.64 μg/mL, and 63.83 μg/mL, respectively, implying their therapeutic potential. The five publications on this subject matter encompass an exceedingly comprehensive array of data. The investigation conducted by Sousa et al. (2019) [118] disclosed the anti-Leishmania infantum activity of 48 seaweed extracts from the Iberian coast, with extracts from *Cystoseira baccata* (*Gongolaria baccata*), *Cystoseira barbata* (*Treptacantha barbata*), *Cystoseira tamariscifolia* (*Ericaria selaginoides*), *Cystoseira usneoides*, *Dictyota spiralis*, and *Plocamium cartilagineum* demonstrating promising activity. This observation suggests the potential of Sargassaceae macroalgae as sources of antileishmanial compounds. In a subsequent investigation, de Sousa et al. (2017) [119] isolated and characterized two antileishmanial meroditerpenoids from the alga *Cystoseira baccata* (*Gongolaria baccata*), displaying significant activity against promastigotes and amastigotes of Leishmania infantum. Simultaneously, Ainane et al. (2014) [120] examined the antibacterial, antileishmanial, and cytotoxic activities of extracts from the alga *Cystoseira tamariscifolia* (*Ericaria selaginoides*), emphasizing the remarkable biological activity. A study conducted by Oliveira et al. (2014) [121] assessed the antileishmanial activity of extracts from *Cystoseira baccata* (*Gongolaria baccata*) and *Cystoseira barbata* (*Treptacantha barbata*), accentuating the impact of the extraction method on this activity, with extracts obtained through hot extraction exhibiting higher activity. Lastly, the study by Mohamed Abdoul-Latif et al. (2021) [122] explored the synthesis of silver oxide nanoparticles from *Cystoseira amentacea* (*Ericaria amentacea)* extracts, unveiling noteworthy antileishmanial activity and presenting promising prospects for the treatment of leishmaniasis.

The exploitation of Sargassaceae algae, particularly of the genera *Cystoseira* and *Bifurcaria*, as a therapeutic alternative in the treatment of leishmaniasis represents an innovative and diversified approach within the medical landscape. The encouraging results suggest the need to persevere in investigations in order to improve efficiency, optimize selectivity, and detail the mechanisms of action specific to algal compounds. These continued efforts could potentially catalyze the development of alternative treatments based on by-products, whether biomolecules, nanoparticles, or engineered formulations, aimed at increased efficacy and better tolerability. This approach would thus contribute significantly to the therapeutic arsenal against leishmaniasis, introducing remarkable sophistication into the medicinal approach to this pathology.

## 4. Conclusions

Leishmaniasis, an affliction of the tropics that has been largely disregarded, is acknowledged by various international organizations as a pressing concern in the realm of global health. The failure to accurately diagnose leishmaniasis in Africa through traditional approaches has resulted in prolonged chemotherapy delays and, ultimately, fatalities in areas highly affected by the disease. Consequently, the precise and prompt identification of the ailment assumes a pivotal role in discerning infected patients, cases involving co-infection, and distinguishing between individuals who are healthy and those who have recovered. Nevertheless, recent diagnostic methodologies, including advanced molecular techniques, flow cytometry, proteomics, and nanodiagnostics, have proven to be groundbreaking in the prognosis of this perilous infection. Similarly, in order to counteract the emergence of drug resistance to conventional therapies, novel strategies have yielded successful outcomes in the treatment of leishmaniasis. This comprehensive overview also encompasses an exploration of natural products, particularly brown algae from the Sargassaceae family. Consequently, scientific data pertaining to the latter are examined, specifically a patent and five articles that describe the antileishmanial properties of products extracted from various species of algae within the genera *Cystoseira* and *Bifurcaria*. These studies provide new and innovative perspectives for the pharmaceutical exploitation of Sargassaceae algae by-products in the battle against leishmaniasis.

## Data Availability

Data are contained within the article.
